# Challenges and Strategies for Pakistan in the Third Wave of COVID-19: A Mini Review

**DOI:** 10.3389/fpubh.2021.690820

**Published:** 2021-08-13

**Authors:** Kashif Kamran, Abid Ali

**Affiliations:** ^1^Department of Zoology, University of Balochistan, Quetta, Pakistan; ^2^Department of Zoology, Abdul Wali Khan University Mardan, Khyber Pakhtunkhwa, Pakistan

**Keywords:** pandemic, COVID-19, outbreak, death, lockdown

## Abstract

The world is currently gripped by the fear of the corona virus disease 2019 (COVID-19) pandemic. The causative agent of COVID-19 is a novel coronavirus known as Severe Acute Respiratory Syndrome Coronavirus 2 (SARS-CoV-2) that attacks humans without prejudice, and primarily targets the respiratory system. Pakistan is a developing country with a large population and a weak economy. Currently, it is facing a major challenge to cope with the outbreak of the COVID-19 pandemic, especially the third wave. This fatal virus has increased its presence many folds in Pakistan. On average, 100 deaths per day were being recorded in the late spring of 2021. Delay in the acquisition of vaccine has slowed down the vaccination program for this disease. This in turn will accelerate the spreading of virus, and thus will lead to a lockdown situation.

**Graphical Abstract:**
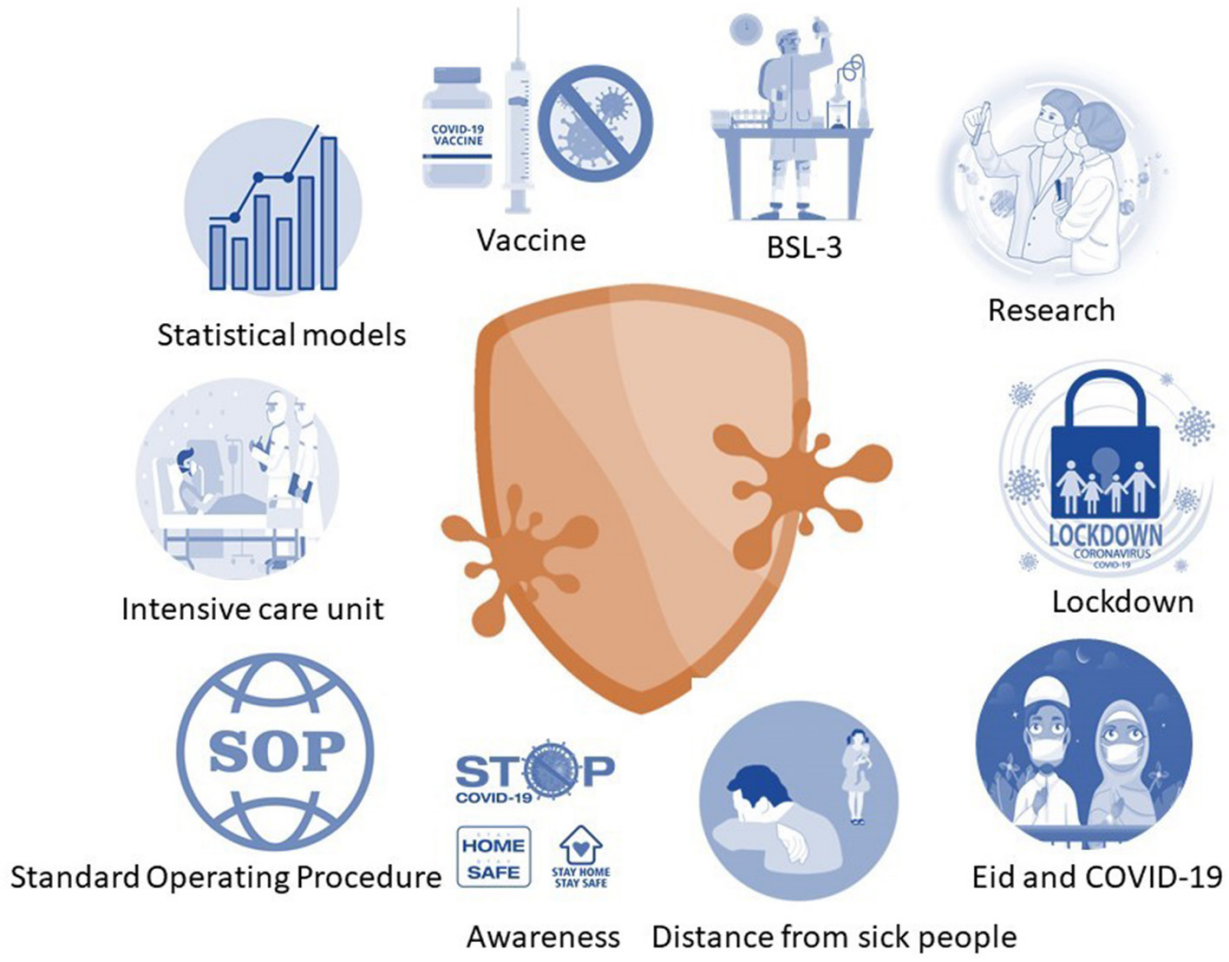
Possible potential approaches for decreasing the risks of COVID-19.

## Origin and History of COVID-19 in Pakistan

The first novel SARS-CoV-2 infected patient was reported in December 2019 (1), and the disease was shortly after named COVID-19 in January 2020 (2). The deadly disease has rapidly spread to around 215 countries since its origin from a seafood wholesale market in Wuhan, a central city in the People's Republic of China (PRC) (3). People would have never imagined that the situation shown in Steven Soderbergh's pandemic thriller movie Contagion (released in 2011) would become reality with the COIVD-19 pandemic (4). There has been considerable discussion on the multiple waves of this causative virus and its control measures.

**Figure 1:**
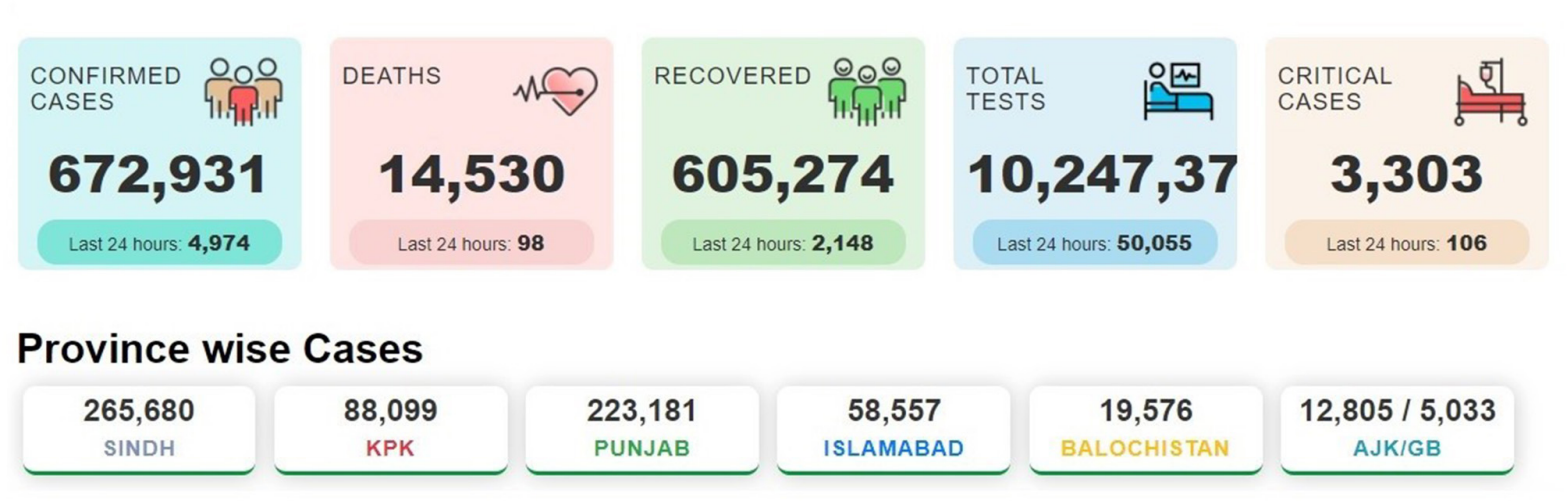
COVID-19 status in Pakistan. The figure illustrates the deaths, confirmed cases, and tests performed from May 2020 to 31st March 2021 (The data were obtained from https://covid.gov.pk/).

**Figure 2:**
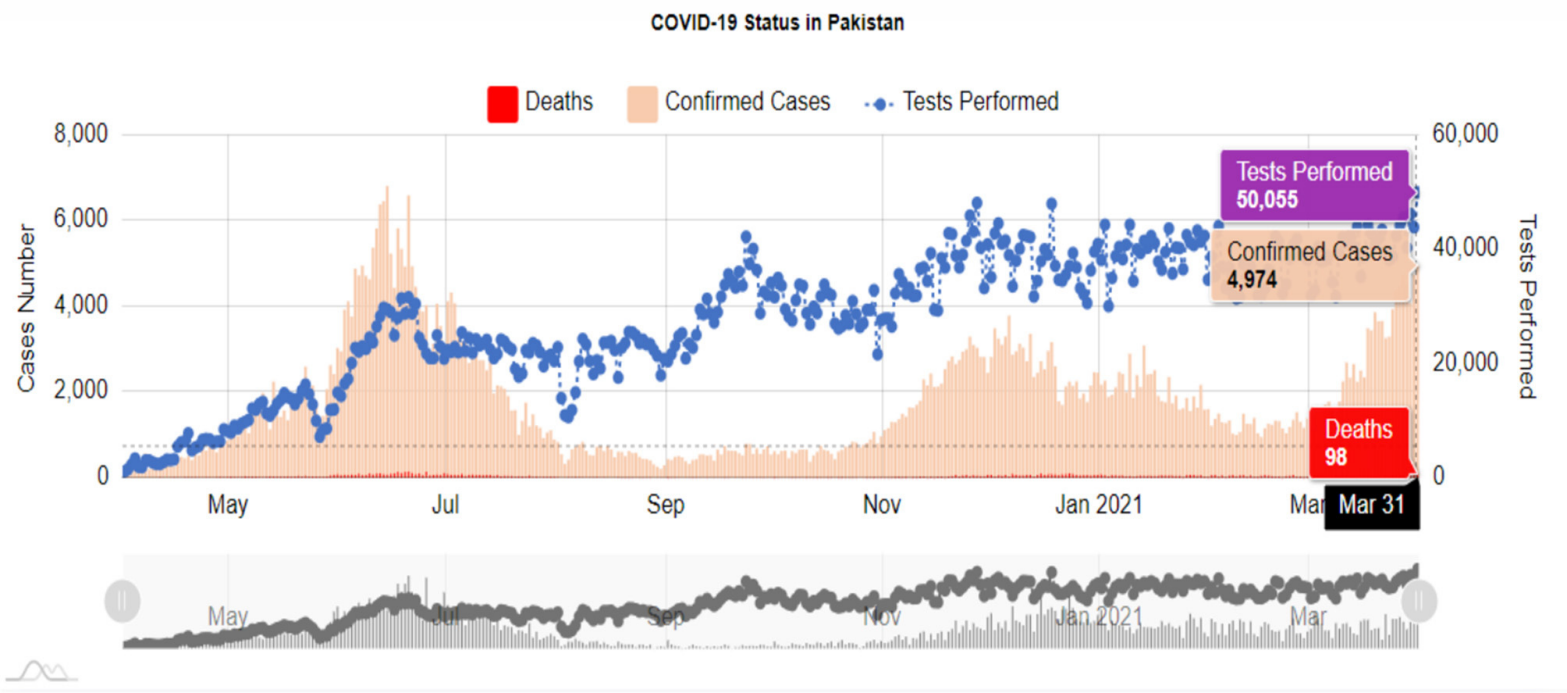
Report of daily deaths due to Covid-19 during March 2021. Values show an exponential growth pattern since its outbreak on 26th February 2020 (data taken from the Pakistan statistics of NCOC, https://covid.gov.pk/).

Pakistan is an under-developed country with a population of about 220 million. It has five provinces: Balochistan, Sindh, Khyber Pakhtunkhwa (KPK), Punjab, and Gilgit Baltistan. The first case of COVID-19 was confirmed on 26th February 2020 by the Ministry of Health, Pakistan (5) and then a continuous spreading of this disease was observed across the country. This virus initially entered Pakistan through returning pilgrims from Iran (through the Taftan border), Saudi Arabia (6), and from Pakistanis who were trapped in other countries that were brought in on special flights (7). Pakistan is currently in a state of health emergency. A total of 1,024,737 tests were carried out with 672,931 (about 66%) confirmed cases of COVID-19 and more than 14,530 (about 2.1%) deaths, reported till late March 2021. Pakistan also experienced the recovery of 605,274 patients (about 90%) and the number of currently reported patients that are under treatment in the health sector is ~53,127 (Figures 1, 2). According to available data, about 300 medical workers in Pakistan are among the fatal victims of COVID-19. The provincial government of Sindh province was the first to implement a full lockdown, due to which the spread of this virus was reduced to some extent.

## First and Second Wave of COVID-19

Since its eruption, the first wave of the disease was very uncertain due to the unknown nature of the virus, its mechanism of infectivity, transmission, and possible treatment options. However, the rapid response of every country, including Pakistan, controlled the fatal prevalence of COVID-19. During the initial wave, Pakistan was very successful in managing the transmission of the disease. It was made possible due to implementation of partial and smart lockdown policies. In partial lockdown, limited time for movement was allowed to citizens after which free movement was strictly prohibited. In smart lockdown, specific areas within a city were sealed where COVID-19 positive cases were detected. Nevertheless, a second wave of the disease occurred, which was moderate in its transmission and pathogenicity. It could have been due to the progressive development in treatment and vaccinations.

## The Third Wave of COVID-19

WHO had already warned the Pakistani government that the number of people infected with COVID-19 could exceed 0.2 million by mid-July, 2020 (8). However, no such expected infection rate was reported. A new variant of SARS-CoV-2 (known as 20I/501Y.V1, VOC 202012/01, or B.1.1.7) emerged from the United Kingdom (9), and has been detected in over 64 countries as of January 27, 2021, including Pakistan (10). This B.1.1.7 variant is associated with an increased risk of death compared to other variants with average deaths of 100 patients reported on a daily basis in Pakistan. Due to the arrival of this new variant, the 10 cities of Pakistan, Bahawalpur, Faisalabad, Hyderabad, Islamabad, Lahore, Multan, Muzaffarabad, Peshawar, Rawalpindi, and Swat, were put under stiff lockdown till April 11, 2021, where the provincial administration was directed to observe the strict implementation of SOPs.

One thing that was very common between the first and third wave was the time of onset i.e., Spring (from March till end of April). It may lead to the generation of a hypothesis that pollens have a significant role in the enhanced transmission of SARS-CoV-2 virus.

## COVID-19 and Comorbidities

It has been observed in several cases that COVID-19 patients develop certain additional comorbidities like typhoid, Myocarditis, blood coagulation, and the fatal attack of black fungus. The co-occurrence of these secondary disorders clearly indicate that SARS-CoV-2 provides a favorable condition for the growth of other microbes, however, its mechanism of action is still unexplored.

## Drugs and Vaccination Programs

As of June 17, 2020, there was no vaccine available for COVID-19, but various drugs i.e., Tocilizumab, Bemsivir lyophilized, Ninavir lyophilized, Dexamethasone and Azithromycin, were used to treat the disease without proper regulation. Therefore, the only way to stop the spread of the virus was to observe self-isolation and social distancing from other people. The Drug Regulatory Authority of Pakistan (DRAP), after taking into account the set standards of quality and safety, approved hydroxychloroquine as the first medicine to treat COVID-19 patients. Later on, convalescent plasma therapy was also approved for the treatment of seriously affected COVID-19 patients. Then, a combination of the drugs chloroquine and azithromycin was also found to be effective in clinical trials against COVID-19 (11). Tocilizumab, an anti–interleukin-6 receptor monoclonal antibody, was also successful in a trial at Agha Khan Hospital, Karachi, Pakistan (12), however this method was not adopted by the Federal Government as a possible treatment to cure COVID-19 patients.

**Figure 3:**
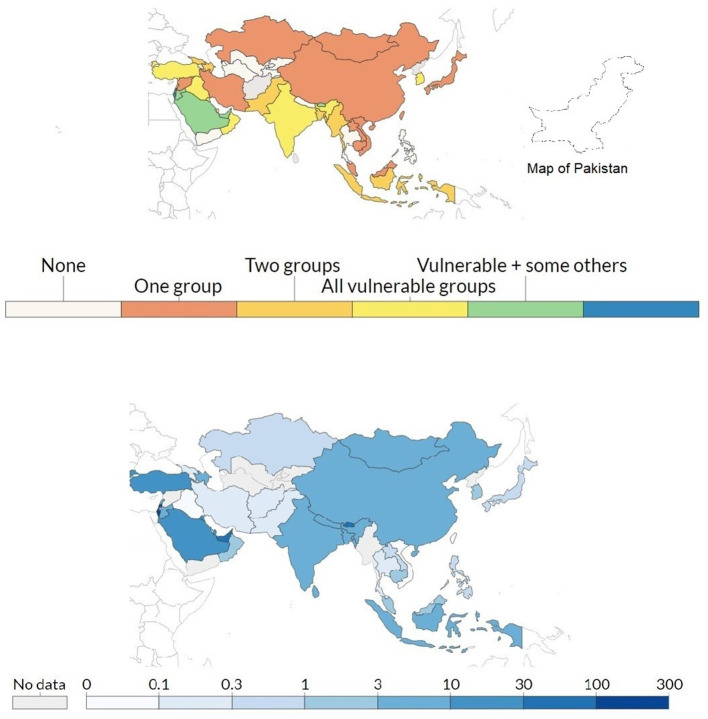
**Top**: Metric records policies in Asian countries for vaccine delivery for three groups: (i) keyworkers, (ii) clinically vulnerable, and (iii) the elderly. Here one means to ensure the supply of the vaccine to one of the three groups, two means to ensure that two of three groups receive the vaccine, while three means ensuring that all groups receive the vaccine equally. **Bottom**: Metric record of the total number of vaccination doses administered per 100 people in the total population. This data counted as a single dose, even though people have received two doses of the vaccine (opensource: https://ourworldindata.org/).

Today, vaccines are widely used to protect millions of people around the world from various infectious diseases. Therefore, it was expected that this pandemic would be controlled after the development of a corona vaccine. In this hope, Russia announced the first relevant vaccine (Sputnik-V) within just 15 weeks of the outbreak of the corona virus (13). Presently, DRAP has approved the four most promising vaccines: CanSino (CanSinoBIO, China), Sinopharm (Sinopharm Group Co., Ltd, China), Sputnik-V (Gamaleya Research Institute of Epidemiology and Microbiology, Russia), and Oxford-AstraZeneca (a joint adventure of the University of Oxford and Vaccitech Limited, United Kingdom). The vaccine developed by Pfizer was not approved due to its extreme low preservation temperature (i.e., −80°C) as Pakistan lacks such facilities. The above stated four vaccines can be preserved around 0°C, which is quite easy to manage. Pakistan had received a donation of 3 million vaccine doses by mid- April, 2021 from CanSino. Pakistan will also receive 17 million doses of Oxford-AstraZeneca vaccine under the WHO-led COVAX program for developing countries (14). According to a Gallup Survey Poll of Pakistan, “*Sixty percent of Pakistanis said that they would get the COVID-19 vaccine if it became available, whereas 34% respondent were worried about the side effects and 22% are against the vaccine*” (15). The hallmarks of COVID-19 is that it has a higher mortality and pathogenicity in elderly people as compared to those younger than 18 (16). Currently, frontline health care workers and senior citizens (above 60 years) are receiving the Sinopharm vaccine (Figure 3) which consists of two doses and has been donated by China. The Federal Government has also issued licenses to pharmaceutical companies to purchase the Russian and Chinese vaccines and allowed them to sell to the private sector at a government approved price.

## Fundamental Challenges of COVID-19 in Pakistan

The current pandemic was not treated in Pakistan in the same way as other countries. Earlier predictive models suggested that the onset of summer may reduce the severity of the virus. However, the results of recently conducted research on COVID-19 does not support the earlier model and concluded that this virus is independent of weather conditions and climate change (17). A vast majority of the people did not follow the instructions and guidelines set by the Federal Government and subordinate departments (18) including the National Command Operation Center (NCOC), Ministry of Health, National Disaster Management Authority (NDMA), and the Drug Regulation Authority (DRA), which has resulted in the major spread of COVID-19 in Pakistan.

Despite various efforts made by NCOC to control COVID-19 transmission in Pakistan, some major shortcomings contributed to the deterioration of the corona situation. These include lack of public awareness at rural areas and a non-cooperative approach by the public toward free testing facilities provided by the government to avoid COIVD-19. The number of issues related to COVID-19 are continuously amplifying and getting more complicated each day, leading to the situation being seen as a catastrophic failure by the government (19).

## World Approach During the Pandemic

During the pandemic, the world has learned some important lessons. The first lesson was that health, economy, and human development are interlinked. It is necessary that these parameters be connected to achieve UN sustainable development goals in a coordinated manner (20, 21). It should be emphasized that public health is not an individual or government matter but it is the overall responsibility of a society (22). The third key point is the importance of the development of telemedicine training courses (23), forensic training (24), and patient referral systems (25) for medical professionals and health workers. Most developed countries have developed several statistical evidence based decision-making systems (26) and this should also be extended to under-developed and non-developed countries. It was also observed that the health care system of America collapsed during corona the pandemic despite being the best health care systems in the world. Each country must improve their current ability to deal with emergencies under normal circumstances (27).

## Possible Potential Solutions

The following strategic solutions are proposed to overcome the issue of COVID-19. These solutions are:

- Statistical model-based forecasting tools should be developed that may help in forecasting the spread, recoveries, and deaths from the current outbreak of COVID-19 (28).
- The government must increase the capacity of intensive care units equipped with ventilators and related equipment. At present, only 1,500 ventilators are available throughout the country, which is not enough to cope with the emergent situation. Similarly, only one doctor is available for every 1,720 patients. This overburdens doctors who are treating COVID-19 patients in addition to their normal patients.
- There is a need to develop consensus-based national standard operating procedures (SOPs) to control the COVID-19 situation following the guidelines of the World health organization (WHO) and Center of Disease Control (CDC).
- A campaign should be launched to create more awareness among people regarding the use of face masks and hand wash (29), and avoiding close contact with infected people (30).
- The SOPs, recommended by the Government during the month of Ramadan and Eid, must be strictly followed (31).
- Currently, only 20 biosafety laboratories are operational in Pakistan, and these have a testing capability of 60,000 tests per day. The existing number of biosafety containment level 3 laboratories (BSL-3) require an additional 15 laboratories in order to achieve a target of 100,000 tests daily.
- The government must announce a price limit for corona vaccines for the private sector and it should be as low as possible. It may be pointed out that many developed countries have already fixed the price of the vaccine in their countries (32).

## Conclusion

The virus will continue to spread all over the world till a vaccine is available for each individual. Experts believe that the virus may remain within us for many years. The human race has never experienced such a situation before. All countries of the world should unite to adopt a common strategy under the umbrella of the United Nations platform to control the disease. We must create awareness among people to observe social distancing and lockdowns and wear face (in areas of high prevalence). Serious efforts should be made at the government level without any delay to acquire vaccines and make them available to each person.

## Author Contributions

The topic was devised by KK under the conceptualization and supervision of AA. KK used PubMed for literature review. All authors have read and agreed to the published final version.

## Conflict of Interest

The authors declare that the research was conducted in the absence of any commercial or financial relationships that could be construed as a potential conflict of interest.

## Publisher's Note

All claims expressed in this article are solely those of the authors and do not necessarily represent those of their affiliated organizations, or those of the publisher, the editors and the reviewers. Any product that may be evaluated in this article, or claim that may be made by its manufacturer, is not guaranteed or endorsed by the publisher.
